# Regional Disparities in PCV13 Coverage in Rural Peru

**DOI:** 10.51894/001c.144119

**Published:** 2025-09-30

**Authors:** Dana Simon

**Affiliations:** 1 College of Osteopathic Medicine Michigan State University

## 37

### INTRODUCTION

Streptococcus pneumoniae (S. Pneumoniae) is a gram positive bacteria, known as pneumococcus, and it is the causative bacterial pathogen of pneumonia in pediatrics. This paper provides an analysis of S. Pneumoniae serotypes in indigenous populations, and its comparison to urban populations of Peru, discussing the importance of morbidity and mortality among vaccinated and unvaccinated patients.

### OBJECTIVES/HYPOTHESIS

The objective of this study is to determine the most common S. Pneumoniae serotypes in children under age five in Iquitos and Puno, Peru who are vaccinated with PCV13, and the difference in vaccine coverage of the most common serotypes in urban Lima (capital of Peru). We hypothesize that the serotype distribution in Iquitos and Puno, Peru will differ significantly from Lima, Peru, and will reveal geographical disparities in vaccine coverage.

### METHODS

This was a cross sectional study. Serotypes of S. Pneumoniae were collected via Nasopharyngeal swabs from 612 (312 in Puno and 300 in Iquitos) children under age five in rural communities of Iquitos and Puno, Peru. A contingency table was constructed, and chi-square analyses were performed using MATLAB to determine if there is a statistically significant difference in PCV13 vaccine coverage for region specific common serotypes. These were compared to previously published common serotypes in Lima, Peru.

## RESULTS

The most common serotypes in Iquitos compared to Lima showed a significant difference in PCV13 vaccine coverage (p=0.002391, chi-square=9.2222), and between Puno and Lima (p=0.0098233, chi-square=6.6667), indicating regional disparities.

### DISCUSSION/CONCLUSIONS

The most common serotypes in Lima, Peru are 23F, 19F, 19A, 14, 6B, and 5, which are all covered by PCV13. This indicates that Iquitos, which has a large indigenous population, experiences disparities in vaccine coverage compared to its country’s most populated city. In addition, Puno, also showing significant vaccine coverage disparities compared to Lima, has a majority indigenous population, and most of its citizens live in extreme poverty with little access to education and healthcare. This study explains the significant healthcare disparities that indigenous and rural populations in Peru face, and the need for future studies on region specific vaccination strategies.

